# The Anti-Osteoarthritis Effect of Medicinal and Edible Homologous Bioactive Components and Chinese Medicinal and Health Food Varieties

**DOI:** 10.3390/nu18183088

**Published:** 2026-09-21

**Authors:** Haibin Wang, Guoliang Zhang, Shuqi Zheng, Yanhong Sun

**Affiliations:** Inner Mongolia Medical University, No. 193 Xinhua Street, Huimin District, Hohhot 010110, China; 19834819507@163.com (H.W.);

**Keywords:** osteoarthritis, homology of medicine and food, medicinal health food, bioactive ingredients, cartilage protection

## Abstract

Osteoarthritis (OA) is a chronic progressive joint disease characterized by articular cartilage degeneration, subchondral bone remodeling, osteophyte formation, and synovitis. It is one of the most common disabling diseases in the middle-aged and elderly population. At present, clinical treatment is mainly based on relieving symptoms, and there is still a lack of interventions that can prevent or reverse the disease process. In recent years, bioactive components derived from medicine–food homology (MFH) and Medicinal Health Food (MHF) have received extensive attention in the prevention and treatment of osteoarthritis due to their high safety and multi-target effects. In this paper, bioactive components with anti-osteoarthritis effects in medicinal and edible homologous and medicinal health foods are systematically reviewed, including polyphenols, flavonoids, terpenoids, alkaloids, and polysaccharides. The progress of research into cartilage protection through the regulation of inflammatory signaling pathways, mitigation of oxidative stress, regulation of chondrocyte metabolism, and epigenetic modification is summarized, and the future research directions in this field are discussed.

## 1. Introduction

Osteoarthritis is a chronic progressive joint disease characterized by articular cartilage degeneration, subchondral bone remodeling, osteophyte formation, joint space narrowing, and synovitis [1]. Its clinical manifestations mainly include severe joint pain, stiffness, joint contracture, muscle atrophy, limited mobility, swelling, tenderness, and varying degrees of local inflammation. The pathogenic factors of osteoarthritis are complex and diverse, including genetic susceptibility, eating habits, obesity, gender, aging, joint trauma, mechanical stress, metabolic diseases, and sedentary lifestyles [2].

With the acceleration of the aging process of the global population, the prevalence of osteoarthritis continues to rise and has become one of the major global public health problems. According to the Global Burden of Disease Study 2021, there are about 453.56 million adults aged 55 and over in the world with osteoarthritis in 2021, and the age-standardized prevalence rate (ASPR) is 30,395.11/100,000 population. The number of new cases reached 23.86 million, and the age-standardized incidence rate (ASIR) was 1610.95/100,000. By 2050, the number of osteoarthritis cases is projected to increase by 74.9% to about 106.6 million; the global ASPR will increase by 14%, the ASIR will increase by 9%, and the age-standardized DALY rate (ASDR) will increase by 12% [3,4]. In China, the prevalence of symptomatic knee osteoarthritis in people aged 60 and over is about 8.1%.

At present, the drug treatment of osteoarthritis mainly includes acetaminophen, non-steroidal anti-inflammatory drugs (NSAIDs), tramadol, and opioids. Although these drugs can relieve pain and inflammation to a certain extent, they cannot prevent, reverse, or cure osteoarthritis. More importantly, long-term use of these drugs is often accompanied by adverse reactions such as gastrointestinal injury and cardiovascular risk [5]. In addition, osteoarthritis symptomatic slow-acting drugs (SYSADOAs) such as glucosamine sulfate and chondroitin sulfate are widely used as joint nutritional supplements. However, their clinical efficacy is still controversial; although some studies have shown that they may provide moderate pain relief and functional improvement [6] meta-analyses and clinical guidelines in recent years have questioned their disease modification effects and structural protection benefits [7]. Therefore, finding safe and effective new intervention strategies that can delay the progression of the disease has become an important direction in the field of osteoarthritis research [8].

The concept of “homology of medicine and food” originated from traditional Chinese medicine. The discussion of “consumed on an empty stomach, it is food; consumed by the sick, it is medicine” in the ancient Chinese medical text *Huangdi Neijing Taisu* profoundly reflects this idea [9,10]. At present, 110 kinds of medicinal and edible homologous varieties and 114 kinds of medicinal and health food varieties have been published in China [10]. These substances have the dual functions of nutritional health care and disease prevention. In recent years, a large number of studies have shown that bioactive components derived from medicine and food homology and medicinal health foods have shown multi-target therapeutic potential in the treatment of osteoarthritis. In addition to their well-known anti-inflammatory and antioxidant properties, these compounds have been shown to regulate chondrocyte metabolism, inhibit cartilage matrix degradation, promote extracellular matrix synthesis, regulate subchondral bone remodeling, and even affect epigenetic modification and gut–joint axis homeostasis [11,12,13]. These pleiotropic effects provide new ideas and candidate substances for the prevention and treatment of osteoarthritis.

This article aims to systematically review bioactive components with anti-osteoarthritis effects and their mechanism of action in drug and food homologous and medicinal health foods, in order to provide a reference for further research and application development in related fields.

## 2. Pathological Mechanism and Treatment Status of Osteoarthritis

### 2.1. Core Pathological Features of Osteoarthritis

The core pathological change of osteoarthritis is the progressive degeneration of articular cartilage. Chondrocytes are the only cell type in articular cartilage and are responsible for maintaining the balance of synthesis and degradation of the extracellular matrix [14]. In the pathological process of osteoarthritis, this balance is broken—chondrocyte anabolism is weakened and catabolism is enhanced, resulting in overexpression of matrix metalloproteinases (MMPs) and disintegrin and metalloprotease-containing thrombospondin motifs (ADAMTS), which in turn causes the degradation of type II collagen and proteoglycans [15,16]. At the same time, abnormal remodeling of subchondral bone, osteophyte formation, and chronic inflammatory response of synovium together constitute a complex pathological network of osteoarthritis [17].

### 2.2. The Role of Inflammation and Oxidative Stress in Osteoarthritis

Inflammatory response and oxidative stress play a central role in the development of osteoarthritis. Studies have confirmed that when articular cartilage is in a state of high oxidative stress, it is easy to cause a cellular inflammatory response, leading to tissue damage [18]. Interleukin-1β (IL-1β) and tumor necrosis factor-α (TNF-α) are key cytokines that drive the production of inflammatory mediators and matrix-degrading enzymes in osteoarthritis [19]. These pro-inflammatory factors induce chondrocytes to produce more inflammatory mediators and degrading enzymes by activating nuclear factor-κB (NF-κB), mitogen-activated protein kinase (MAPK), and other signaling pathways, forming a vicious cycle [20,21].

### 2.3. Limitations of Existing Treatment

**Figure 1:**
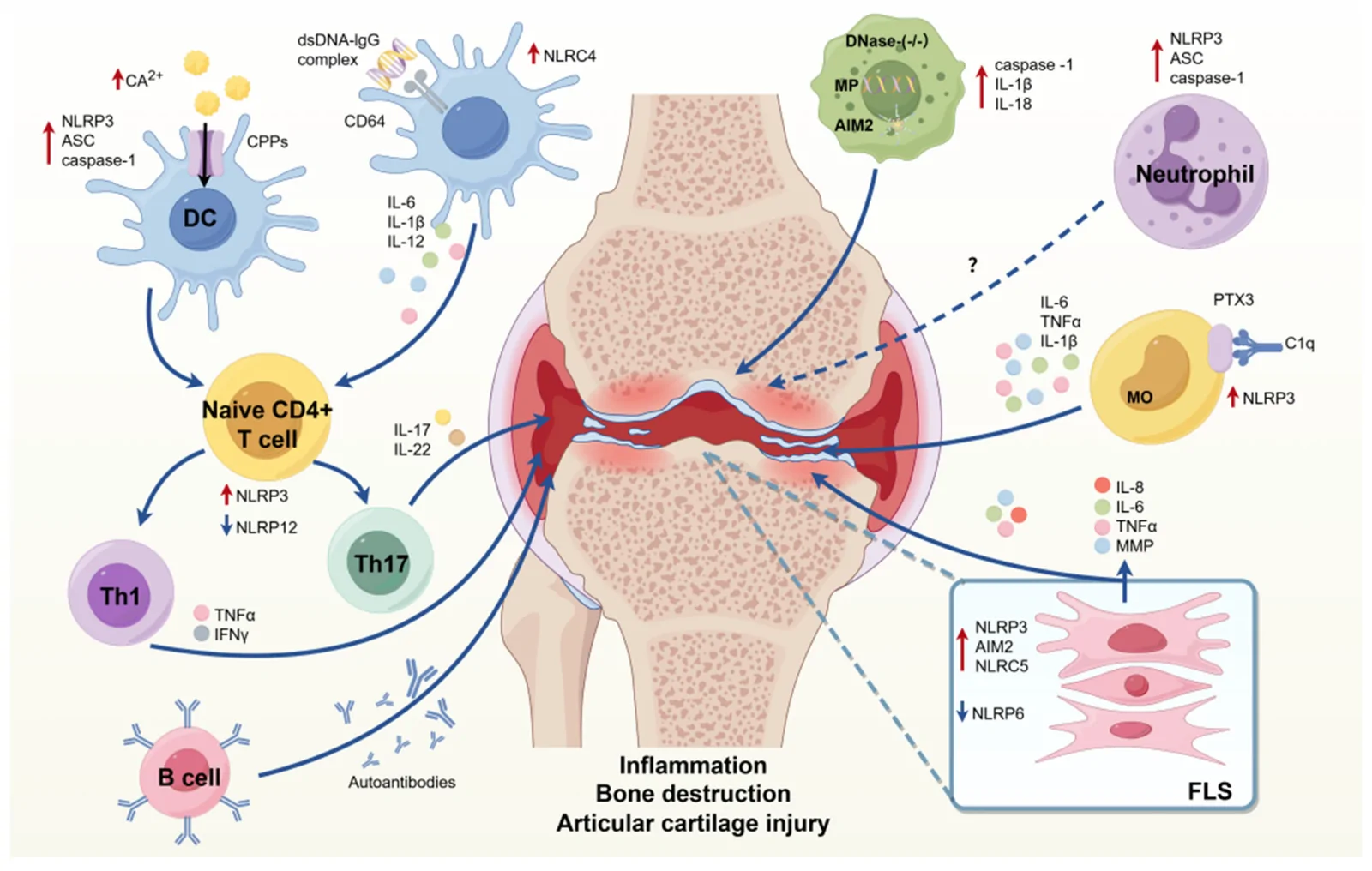
The pathogenesis and pathological changes of osteoarthritis (OA). (Note: ‘?’ represents a pathway that has not been fully elucidated. ➡ represents a well-confirmed pathway. ⇢ represents the pathway that has not been fully confirmed.)

Although non-steroidal anti-inflammatory drugs, corticosteroids, anti-rheumatic drugs, and biological agents can provide symptom relief and immunosuppression for patients with osteoarthritis, their long-term use is often limited by adverse reactions and inability to achieve structural repair. At present, there is no clinical intervention that can truly delay or reverse the progression of osteoarthritis. Therefore, it is of great clinical significance to develop new intervention strategies with both safety and disease modification [5] (Figure 1).

## 3. Anti-Osteoarthritis Active Ingredients of Medicinal and Edible Homologous and Medicinal Health Food

**Figure 2:**
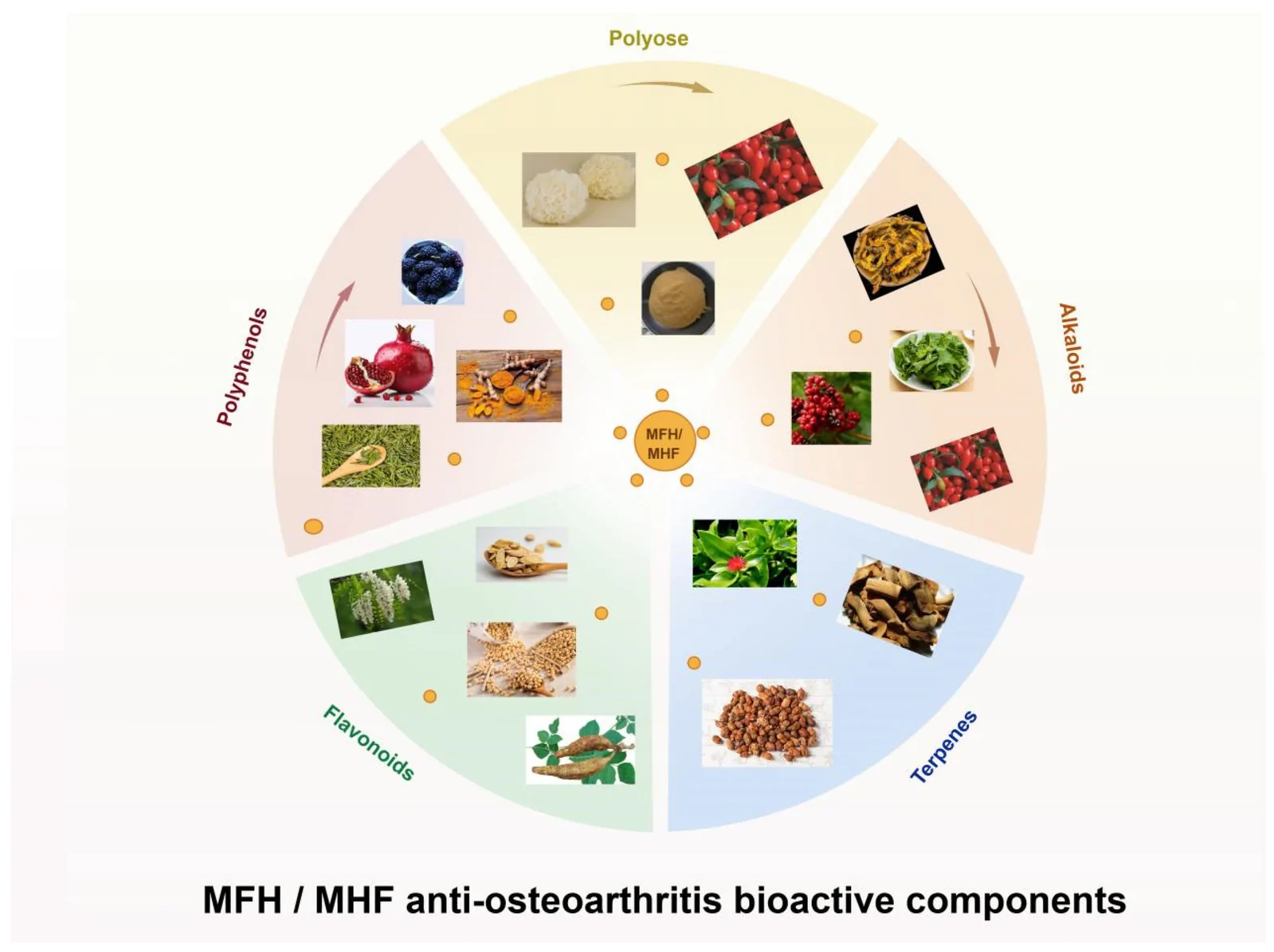
Classification of bioactive components with anti-osteoarthritis effect in MFH/MHF.

Medicinal and edible homologous and medicinal health foods are rich in bioactive components. These plant secondary metabolites can play a role in cartilage protection by inhibiting the expression of inflammatory and catabolic mediators, regulating epigenetic modification, and other ways [22]. However, these bioactivities are highly compound-specific, and caution is needed when extending results from one bioactive compound to the whole group (Figure 2, Table 1).

### 3.1. Selection Criteria and Methodological Considerations

To ensure scientific rigor, clinical relevance, and structural representativeness, this review adopted explicit inclusion criteria for bioactive compounds: (i) the parent substance must be listed in China’s official medicine–food homology (MFH) catalog or Medicinal Health Food (MHF) directory; (ii) the compound must have in vitro or in vivo evidence of anti-osteoarthritic (anti-OA) activity published in peer-reviewed journals; (iii) the molecular mechanism should be partially elucidated at the signaling-pathway or target level; and (iv) priority was given to compounds with available clinical data or those that represent distinct structural scaffolds within their chemical class. Systematic retrieval was performed across PubMed, Web of Science, and CNKI using combinations of “osteoarthritis,” “chondrocyte,” “cartilage degradation,” and specific compound/plant names. Representative compounds were ultimately selected based on the volume and quality of mechanistic evidence, their capacity to illustrate structure–activity relationships (SARs), and their translational potential. Consequently, this review does not claim exhaustive coverage of all MFH/MHF constituents; rather, it focuses on paradigmatic examples that best demonstrate how distinct chemical classes modulate OA pathogenesis.

According to the different chemical structures, representative active ingredients investigated to date can be divided into the following.

### 3.2. Polyphenol Compounds

**Figure 3:**
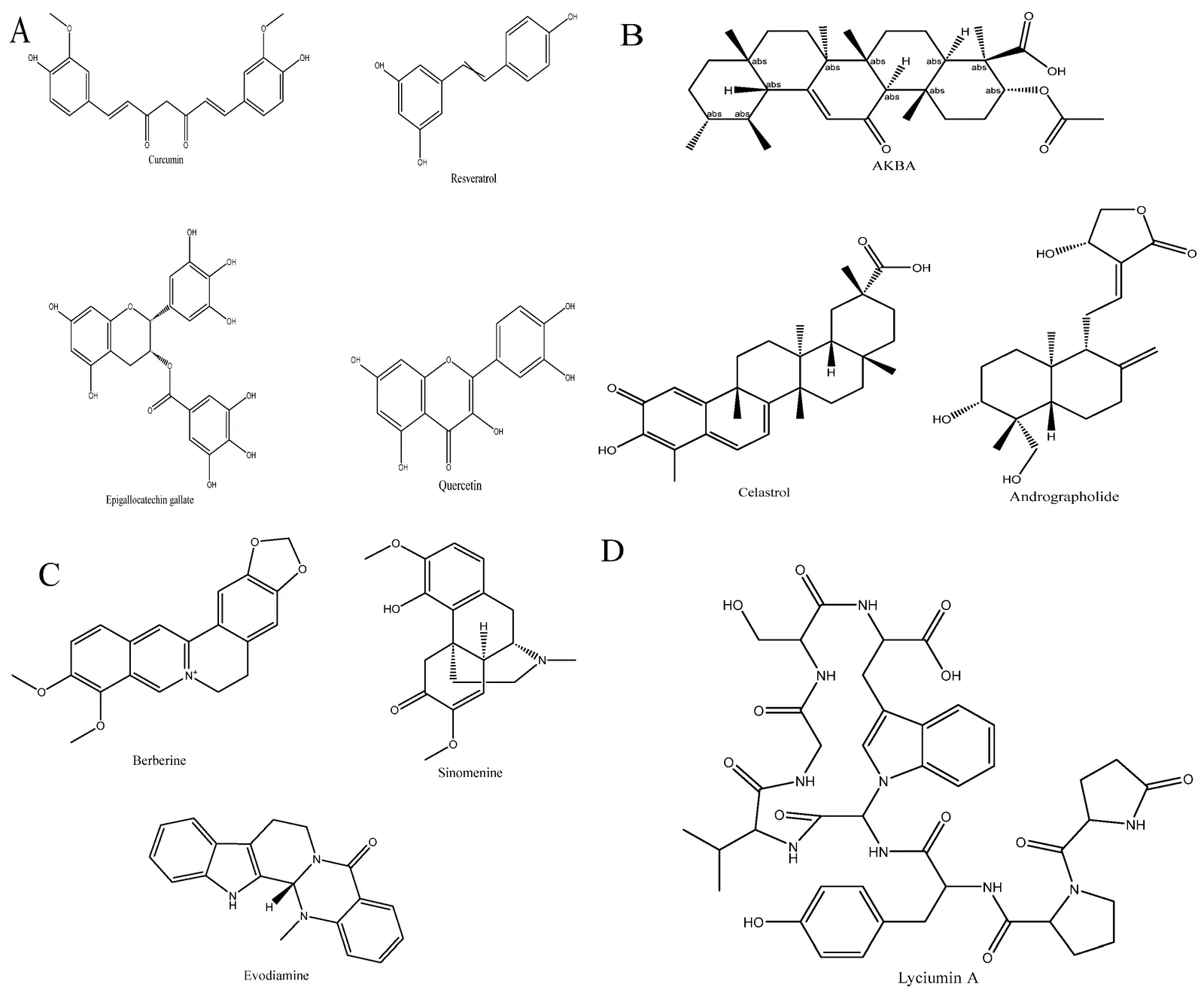
Chemical structure of representative anti-osteoarthritis compounds in MFH/MHF. ((**A**) Polyphenol representative compounds; (**B**) Terpenoids representative compounds; (**C**) Alkaloid representative compounds; (**D**) Polysaccharide representative compounds).

Polyphenols are one of the most widely studied anti-osteoarthritis active ingredients in medicinal and edible health foods [52]. Their therapeutic potential is intimately linked to the number and spatial arrangement of phenolic hydroxyl groups, which confer both antioxidant capacity and the ability to modulate redox-sensitive signaling cascades [53]. This section focuses on three structurally distinct polyphenols—curcumin, resveratrol, and epigallocatechin gallate (EGCG). Because they represent diarylheptanoid, stilbenoid, and flavan-3-ol scaffolds, respectively, and each possesses a unique mechanistic fingerprint in OA [25] (Figure 3A).

#### 3.2.1. Curcumin

Curcumin, the principal diarylheptanoid of *Curcuma longa* L., exhibits a symmetric β-diketone structure with two phenolic rings. This configuration enables it to scavenge free radicals directly and to chelate transition metal ions, but its anti-OA efficacy stems primarily from multi-target signaling modulation. Studies have shown that curcumin can reduce the inflammatory injury of chondrocytes by inhibiting the NF-κβ signaling pathway and the expression of inflammatory factors [23,54]. A standardized turmeric extract has been shown to increase the expression of collagen and aggrecan in articular cartilage of rats with sodium iodoacetate-induced osteoarthritis, while reducing knee joint swelling and cartilage degradation, as confirmed by animal experiments [23,24].

At the molecular level, curcumin covalently binds to cysteine-179 of IKKβ, thereby inhibiting its kinase activity and preventing IκBα phosphorylation and proteasomal degradation [18]. Consequently, nuclear translocation of the NF-κB p65/p50 heterodimer is blocked, leading to downregulation of pro-inflammatory genes including IL-1β, TNF-α, IL-6, and COX-2. Independent of NF-κB, curcumin suppresses the MAPK axis (ERK1/2, JNK, and p38) by interfering with upstream MAPKK phosphorylation, which in turn reduces activator protein-1 (AP-1)-driven MMP transcription [55]. Notably, curcumin also inhibits the NLRP3 inflammasome by preventing ASC oligomerization and caspase-1 autoproteolysis, thereby reducing IL-1β maturation and pyroptotic chondrocyte death. Regarding cartilage anabolism, curcumin upregulates SOX9 expression and promotes type II collagen and aggrecan synthesis while concurrently increasing TIMP-1 to counterbalance MMP-1, MMP-3, and MMP-13 activity [56].

Despite these potent in vitro effects, curcumin’s clinical translation is constrained by poor aqueous solubility (<11 ng/mL), rapid intestinal and hepatic first-pass metabolism, and consequently low oral bioavailability (<1% in plasma). Emerging nano-delivery strategies—including liposomes, polymeric nanoparticles, and phospholipid complexes—are actively being explored to improve its pharmacokinetic profile [57].

#### 3.2.2. Resveratrol

Resveratrol is a polyphenolic compound found in foods such as grapes and peanuts. It is characterized by two phenolic rings connected by an ethylene bridge. Its trans-configuration is crucial for biological activity because the affinity of the cis-isomer to anti-aging enzyme 1 (SIRT1) is significantly reduced. It plays a dual role as a dietary supplement and therapeutic agent under the framework of medicine and food homology: first, as a direct ROS scavenger and phase-II antioxidant enzyme inducer via activation of the Nrf2/Keap1/ARE pathway, and second, as an epigenetic modulator that activates SIRT1, leading to deacetylation of NF-κB p65 and suppression of its transcriptional activity [58]. SIRT1 activation also inhibits the NLRP3 inflammasome and reduces p53-mediated chondrocyte apoptosis. In vivo, resveratrol attenuates cartilage degradation in OA animal models by restoring the balance between MMPs and TIMPs and by reducing subchondral bone sclerosis [25].

Clinical trials have shown that resveratrol can improve bone mineral density and relieve osteoarthritis symptoms [26]. However, the low bioavailability of resveratrol and the difficulty of targeted delivery are still the main challenges restricting its clinical application. After oral administration, resveratrol is rapidly metabolized in the liver and intestines through glucuronidation and sulfation, resulting in minimal systemic circulation of the parent compound, with an oral bioavailability of less than 1% [59,60,61].

#### 3.2.3. Epigallocatechin Gallate (EGCG)

EGCG, the most abundant catechin in *Camellia sinensis*, possesses a characteristic galloyl moiety at the 3-position of the C-ring. This galloyl group is essential for its biological activity, as it facilitates hydrogen bonding with protein targets and enhances membrane permeability. EGCG protects chondrocytes primarily by inhibiting IL-1β–induced NF-κB and AP-1 activation, which suppresses downstream MMP-13 and ADAMTS-5 expression [27]. Additionally, EGCG activates the Nrf2 pathway, upregulating HO-1 and NQO1 to mitigate oxidative stress-induced mitochondrial dysfunction [28]. Its anti-apoptotic effect is mediated through the PI3K/Akt/Bcl-2 axis, where EGCG prevents cytochrome c release and caspase-3 activation [62]. Importantly, the catechol structure of the B-ring allows EGCG to chelate Fe^2+^/Cu^2+^, thereby suppressing Fenton-chemistry-driven hydroxyl radical formation in the joint microenvironment (Figure 2).

### 3.3. Flavonoids

Flavonoids are widely found in fruits, vegetables, and medicinal and edible plants, and are another important class of anti-osteoarthritis active ingredients [63]. Flavonoids share a common C6-C3-C6 skeleton but differ in the oxidation state of the C-ring and the pattern of hydroxylation/methoxylation on the A- and B-rings. These structural variations dictate their anti-OA potency, bioavailability, and target selectivity. This review focuses on quercetin (flavonol), formononetin (isoflavone), and anthocyanins, which exemplify distinct flavonoid subclasses with well-documented chondroprotective effects [54].

#### 3.3.1. Quercetin

Quercetin is a common flavonol compound found in a variety of medicinal and edible plants. Quercetin (3,3′,4′,5,7-pentahydroxyflavone) is distinguished by a hydroxyl group at the 3-position and a catechol moiety on the B-ring. The 3-OH and 4′-OH groups are particularly critical for its antioxidant activity, as they donate hydrogen atoms to neutralize peroxyl radicals and stabilize the resulting aryloxyl radical. Mechanistically, quercetin inhibits IL-1β–stimulated NF-κB nuclear translocation by preventing IκBα degradation and p65 phosphorylation at Ser536 [29]. Simultaneously, it suppresses the MAPK cascade (p38, JNK, and ERK1/2) and downregulates COX-2 and iNOS expression, thereby reducing prostaglandin E_2_ and nitric oxide levels in chondrocytes. Quercetin also activates Nrf2/HO-1 signaling, enhancing endogenous antioxidant defenses.

Studies have shown that quercetin exerts anti-inflammatory and cartilage protection in human OA chondrocytes by suppressing NF-κB nuclear translocation, inhibiting the phosphorylation of IκBα and p65, and attenuating MAPK signaling [30,54].

#### 3.3.2. Formononetin

Formononetin, an O-methylated isoflavone isolated from *Astragalus* and *Glycyrrhiza* species, functions as a phytoestrogen that can bind estrogen receptors (ERα/ERβ) on chondrocytes. Its isoflavone skeleton mimics 17β-estradiol, allowing it to modulate cartilage metabolism via genomic and non-genomic estrogenic pathways. Formononetin attenuates OA progression by downregulating the PTEN/AKT/NF-κB axis and suppressing MAPK phosphorylation, which collectively reduces MMP-3, MMP-13, and ADAMTS-5 expression while preserving type II collagen and aggrecan integrity.

Animal studies demonstrate that formononetin (50 mg/kg, oral gavage) significantly reduces cartilage lesions and synovial hyperplasia in rat OA models, an effect partly reversed by the ER antagonist ICI 182780, confirming receptor-mediated activity [31].

Dietary flavonoids such as anthocyanins (e.g., cyanidin-3-glucoside and pelargonidin-3-glucoside) and flavonols (e.g., quercetin, kaempferol, myricetin) also show positive effects on cartilage protection in osteoarthritis. Pelargonidin ameliorates inflammatory response and cartilage degeneration by suppressing the NF-κB pathway [32]. Myricetin and kaempferol decrease the production of TNF-α, IL-6, NO, and PGE2, suppress COX-2, iNOS, MMP-13, and ADAMTS-5 expression, and inhibit NF-κB signaling in chondrocytes.

### 3.4. Terpenoids

Terpenoids are a kind of natural hydrocarbon compound widely existing in nature that are abundant in medicinal and edible plants. According to the number of isoprene units in the molecule, they can be divided into monoterpenes, sesquiterpenes, diterpenes, and triterpenoids [64]. Their lipophilic nature facilitates membrane permeability and interaction with intracellular lipid-binding domains, making them potent modulators of inflammatory lipid mediators and steroid-responsive pathways. In recent years, important progress has been made in the research of a variety of terpenoids in the prevention and treatment of osteoarthritis (Figure 3B).

#### 3.4.1. Boswellic Acid (AKBA)

Boswellic acid is the main active triterpenoid component in *Boswellia serrata*. It has been used in Indian Ayurvedic medicine and traditional Chinese medicine for thousands of years, and is often used to treat inflammatory diseases and joint pain [33]. 3-O-Acetyl-11-keto-β-boswellic acid (AKBA), the most pharmacologically active triterpenoid in *Boswellia serrata*, possesses a pentacyclic lupane skeleton with an acetyl group at C-3 and a keto group at C-11. These structural features are essential for its dual inhibitory action on 5-lipoxygenase (5-LOX) and the NF-κB pathway. AKBA is a direct, non-redox, non-competitive inhibitor of 5-LOX; it binds to the enzyme’s allosteric site, preventing leukotriene B_4_ and cysteinyl leukotriene synthesis.

Animal experiments have confirmed that AKBA treatment can significantly inhibit the expression of inflammatory cytokines (TNF-α, IL-1β, IL-6) and matrix metalloproteinases (MMP-2, MMP-3, MMP-9) in osteoarthritis models by suppressing NF-κB pathway activation and preventing IκBα phosphorylation and degradation [34]. In a mouse DMM model, AKBA reduced cartilage erosion, synovitis, and osteophyte formation by inhibiting IL-1β/TLR signaling and 5-LOX activity [65]. Clinical studies also provide strong evidence for the anti-osteoarthritis effect of boswellic acid. A number of randomized double-blind clinical trials have consistently shown that patients treated with Indian frankincense extract have significantly reduced knee pain and substantially improved overall activity function [66]. This indicates that frankincense acid can not only alleviate symptoms, but also may have a disease modification effect that delays structural degeneration.

#### 3.4.2. Andrographolide

Andrographolide, a diterpenoid lactone from *Andrographis paniculata*, features an α,β-unsaturated lactone ring and multiple hydroxyl groups. This Michael acceptor functionality allows it to form adducts with cysteine residues on signaling proteins, particularly within the NF-κB and MAPK pathways. Andrographolide suppresses IL-1β and TNF-α production by blocking NF-κB nuclear translocation and inhibiting p38 MAPK phosphorylation.

Studies have shown that andrographolide can improve knee joint function and reduce knee joint swelling, pain, and other symptoms in rats with KOA, as well as cartilage tissue lesions. It is worth noting that andrographolide can interfere with the process of osteoarthritis by regulating the expression of microRNA. Specifically, andrographolide inhibits the pro-fibrotic phenotype of chondrocytes and improves osteoarthritis by up-regulating miR-137, negatively regulating BMP7 and downstream pro-fibrotic markers (COL1A1, COL3A1, α-SMA) [35].

#### 3.4.3. Celastrol

Celastrol, a quinone methide triterpene from *Tripterygium wilfordii*, contains a highly reactive quinone methide moiety that interacts with thiol groups on IKKβ and Keap1. By inhibiting IKKβ, celastrol blocks NF-κB activation; by modifying Keap1 cysteines, it stabilizes Nrf2 and induces antioxidant gene expression. This dual anti-inflammatory and antioxidant action reduces synovial inflammatory infiltration, pannus formation, and cartilage erosion in vivo.

Studies have shown that celastrol can inhibit cartilage destruction and reduce the secretion of pro-inflammatory factors in osteoarthritis. In IL-1β-stimulated chondrocytes and mouse OA models, celastrol attenuates matrix catabolism, oxidative stress, and inflammation by suppressing NF-κB signaling and activating autophagy [36,37].

### 3.5. Alkaloids

Alkaloids are a class of nitrogen-containing alkaline natural organic compounds, which are widely found in the plant kingdom, with diverse chemical structures and significant biological activities. Their alkalinity and ability to cross biofilms give them strong pharmacological activity. The isoquinoline, morphine, and quinolone alkaloids discussed here are selected because they target different nodes in the pathogenesis of OA (Figure 3C).

#### 3.5.1. Berberine

Berberine, an isoquinoline alkaloid from *Coptis chinensis* and *Phellodendron amurense*, carries a tetracyclic structure with a quaternary ammonium center. It has rich sources and good safety. It has important research value and development potential in the treatment of osteoarthritis and other bone- and joint-related diseases.

Studies have shown that berberine can maintain the balance of bone metabolism, inhibit inflammatory response, reduce the production of pro-inflammatory factors, regulate immune metabolism, regulate autophagy and apoptosis, and reduce joint structural damage and pain caused by traumatic osteoarthritis. In vivo studies in collagenase- and surgically induced osteoarthritis animal models have demonstrated that berberine ameliorates cartilage degradation while inducing chondrocyte proliferation via activating AMPK signaling and suppressing p38 MAPK activity [38]. Berberine also decreases inflammation and cartilage degradation by inhibiting TLR4/NF-κB signaling and modulating the autophagy/apoptosis balance in chondrocytes [39,67].

#### 3.5.2. Sinomenine

Sinomenine, a morphinan alkaloid from *Sinomenium acutum*, is structurally related to codeine but lacks significant opioid receptor affinity. Its anti-OA activity stems from inhibition of COX-2 and microsomal prostaglandin E synthase-1 (mPGES-1), coupled with suppression of NF-κB and JAK/STAT3 signaling.

In the study of osteoarthritis, sinomenine hydrochloride can significantly reduce the content of IL-1β and prostaglandin E2 (PGE2) in the synovial fluid of a rabbit osteoarthritis model [40]. In rabbits with cast-immobilization-induced OA, weekly intra-articular injection of sinomenine for 5 weeks significantly reduced IL-1β levels in synovial fluid and serum, leading to improved histological Mankin scores and reduced cartilage degradation [68]. Sinomenine can relieve knee pain, swelling, and morning stiffness in patients with osteoarthritis, and significantly improve knee dysfunction [41].

#### 3.5.3. Evodiamine

Evodiamine, an indoloquinazoline alkaloid from *Evodia rutaecarpa*, inhibits COX-2 expression and suppresses synovial angiogenesis by downregulating VEGF/VEGFR2 signaling. *Evodia rutaecarpa*, a traditional Chinese herbal medicine recorded in ancient and modern Chinese pharmacopeias, contains alkaloids, bitterness, volatile oil, flavonoids, and other active ingredients, and is a medicine–food homology substance.

Studies have shown that evodiamine can decrease the production of NO, IL-6, TNF-α, and PGE2, inhibit the expression of iNOS, COX-2, and MMP-13, and alleviate aggrecan and type II collagen degradation in IL-1β-activated chondrocytes by suppressing NF-κB signaling pathway activation and p65 nuclear translocation [42,43]. In a mouse DMM model, evodiamine alleviated cartilage degeneration and reversed increased OARSI scores [43].

### 3.6. Polysaccharides and Other Components

Polysaccharides are another kind of important bioactive component in medicinal and health food. They are formed by the polymerization of more than 10 monosaccharides through glycosidic bonds. They have various pharmacological activities such as immune regulation, anti-inflammatory, and anti-oxidation (Figure 3D).

*Millettia speciosa* is a well-known medicinal and edible Chinese herbal medicine in China. It has been recorded in Chinese medicine classics that it has the effects of strengthening muscles and bones, tonifying deficiency, and activating collaterals. Network pharmacology analysis showed that the active components of *Millettia speciosa* Champ. (including flavonoids and triterpenoid saponins) may mediate key signaling pathways such as PI3K/Akt, TNF, and IL-17 by regulating core targets such as TNF, IL-1β, and Akt1, and exert anti-osteoarthritis effects through multiple links such as inflammatory response, extracellular matrix (ECM) metabolism, and bone remodeling [69]. Animal experiments confirmed that the ethanol extract of *Millettia speciosa* can reduce the inflammatory response and improve subchondral bone microstructure damage and cartilage damage by blocking PI3K and NF-κB signal transduction and down-regulating MMP-13 expression [70].

*Lycium barbarum* polysaccharide is one of the main bioactive components in *Lycium barbarum*. Studies have shown that LBP can reduce the levels of IL-1β and TNF-α in osteoarthritis chondrocytes, downregulate iNOS expression, and inhibit NF-κB p65 protein expression [44]. *Lycium barbarum* polysaccharide has a strong anti-inflammatory and antioxidant capacity and has a positive effect on bone health. It can inhibit osteoclast-mediated bone resorption and promote bone formation mediated by osteoblasts and bone marrow mesenchymal stem cells [45].

*Eucommia ulmoides* polysaccharide is an important active ingredient in *Eucommia ulmoides*. *Eucommia ulmoides* polysaccharide can significantly reduce the degree of damage to rabbit articular cartilage, increase the density of subchondral cancellous bone, and increase the number and thickness of trabecular bone. Eucommia polysaccharide can also reduce M1-polarized macrophages and increase M2-polarized macrophages, thereby promoting articular cartilage repair and subchondral bone reconstruction [46]. *Eucommia ulmoides* is one of the commonly used and effective traditional Chinese medicines for the treatment of knee osteoarthritis. In addition, tremella polysaccharide also has the effect of inhibiting osteoarthritis, which can protect cartilage tissue and resist apoptosis to a certain extent.

Phytoestrogens also show the potential to delay the degeneration of articular chondrocytes. The proliferation and apoptosis of chondrocytes are most closely related to the formation and development of osteoarthritis. Therefore, delaying the degeneration of articular chondrocytes is particularly important for the prevention and treatment of osteoarthritis. Some traditional Chinese medicines containing phytoestrogens can promote the proliferation of chondrocytes and the stability of their phenotypes, thereby delaying the occurrence of osteoarthritis. In addition to the above-mentioned traditional Chinese medicines, velvet antler, eucommia, *Polygonum cuspidatum*, white peony root, wolfberry, soybean yellow roll, *Astragalus*, licorice, and other traditional Chinese medicines also contain phytoestrogens, which can promote osteoblast proliferation, protect chondrocytes, and regulate cell metabolism. It has been used in the treatment of osteoarthritis and other bone and joint diseases. For example, psoralen, a plant-derived estrogen analogue, can inhibit osteoarthritis through estrogen-like effects [71]. In clinical practice, multiple phytoestrogens are often used in combination to enhance the efficacy.

### 3.7. Medicinal and Edible Compound and Extract

In addition to a single active ingredient, the compound extracts of a variety of medicinal and edible medicinal materials also exhibit anti-osteoarthritis activity. Studies have shown that turmeric extract and pueraria coicis extract can promote the synthesis and secretion of cartilage collagen in osteoarthritis rats and have an improvement effect on joint repair. The single and combined use of turmeric extract and kudzu root coix seed extract can have improve the cartilage injury of knee osteoarthritis rats, mainly by having the synergistic effect of reducing knee joint swelling (inflammation) and promoting chondrocyte repair and joint matrix repair [48].

Jiuwei Jianbu Decoction is a traditional Chinese medicine compound for the treatment of osteoarthritis. Studies have shown that Jiuwei Jianbu Decoction can significantly reduce oxidative stress and inflammatory injury in knee osteoarthritis rats and improve cartilage pathological changes by inhibiting the HMGB1/RAGE/PI3K/Akt signaling pathway [49,51]. In a rat model of knee osteoarthritis, Jiuwei Jianbu Decoction intervention significantly reduced knee joint diameter, improved mechanical and thermal pain thresholds, decreased Mankin and OARSI scores, and suppressed the elevated levels of NO, MDA, TNF-α, and IL-1β in a dose-dependent manner. Mechanistically, it downregulated the expression of HMGB1, RAGE, p-PI3K/PI3K, and p-Akt/Akt in knee joint tissues [50]. As one of the commonly used and effective traditional Chinese medicines for the treatment of knee osteoarthritis, the clarification of its mechanism of action and the material basis of pharmacodynamics is of great significance for wider clinical application. In addition, Xianling Gubao Capsule (XLGB) contains a variety of phytochemicals, including flavonoids, coumarins, saponins, alkaloids, and terpenoids. After oral administration, the active ingredients can strengthen the bones and are used to treat or prevent osteoporosis, osteoarthritis, bone loss, and fracture [72].

With the development of bioinformatics and systems biology, the prediction of traditional Chinese medicine based on the characteristic genes of osteoarthritis has become a new research direction [73,74]. Using bioinformatics analysis based on GEO datasets (GSE55235, GSE169077, and GSE55457) combined with Mendelian randomization, researchers have identified solute carrier family 2 member 3 (SLC2A3) and atypical chemokine receptor 1 (ACKR1) as characteristic genes of osteoarthritis. Through the Coremine Medical and HERB databases, seven traditional Chinese medicines with food–medicine homology properties, including *Evodia rutaecarpa*, *Perilla frutescens* seed, *Ganoderma lucidum*, *Gastrodia elata*, *Prunus armeniaca* seed, *Syzygium aromaticum*, and *Rehmannia glutinosa*, were predicted as potential therapeutic candidates for OA, with β-sitosterol and stigmasterol identified as core active components [68]. Studies have shown that perilla leaf extract has a reversal effect on weight loss and organ index changes in osteoarthritis model mice. It can improve the changes in serum inflammatory factors and matrix metalloproteinase expression levels caused by osteoarthritis, and improve knee joint injury and pathological changes in osteoarthritis mice [75]. *Perilla frutescens* extract has been demonstrated to possess potent anti-inflammatory properties by suppressing the production of NO, ROS, and pro-inflammatory cytokines (TNF-α, IL-1β, IL-6) in LPS-stimulated RAW 264.7 macrophages. It inhibits NF-κB p65 nuclear translocation and downregulates the expression of iNOS and COX-2 in a dose-dependent manner [76]. These predictions provide a new direction for the discovery of anti-osteoarthritis active ingredients in medicinal and edible Chinese medicines (Table 1). In the future, molecular docking and molecular dynamics simulation can be combined to further verify the therapeutic potential of these medicinal and edible Chinese medicines and their core components.

## 4. Mechanism of Anti-Osteoarthritis

The bioactive components derived from MFH and MHF exert anti-osteoarthritis effects through a sophisticated, multi-layered regulatory network. Beyond the traditionally emphasized anti-inflammatory and antioxidant properties, these compounds modulate chondrocyte metabolism, epigenetic landscapes, subchondral bone remodeling, autophagy, ferroptosis, mitochondrial homeostasis, and even gut–joint axis communication. This section provides a comprehensive, mechanistic overview of these pathways and their interconnections. The mechanisms demonstrated for individual representative compounds include the following, but they do not represent all members of the chemical class.

### 4.1. Regulation of Inflammatory Signaling Pathway

Abnormal activation of inflammatory signaling pathways is one of the core links in the pathogenesis of osteoarthritis. Some medicinal and edible bioactive components have been shown to exert anti-inflammatory effects through multi-target regulation of inflammatory signaling pathways [67,77]. However, the specific targets and efficacy of different compounds vary widely.

NF-κB signaling pathway is the core regulatory pathway of the inflammatory response. Curcumin inhibits NF-κB activation in chondrocytes by suppressing IκBα phosphorylation and preventing p65 nuclear translocation, thereby reducing the expression of inflammatory cytokines including IL-1β, TNF-α, and IL-6 [23,78]. Resveratrol attenuates NF-κB signaling and concurrently activates SIRT1/AMPK pathways, which contributes to its suppression of NLRP3 inflammasome activation and pyroptosis in inflammatory cells [79]. Quercetin and its glucosides suppress NF-κB signaling by inhibiting IκBα and p65 phosphorylation, attenuating NF-κB nuclear translocation, and inactivating downstream inflammatory gene transcription in IL-1β-stimulated chondrocytes [54]. Tannic acid, a hydrolyzable polyphenol, can prevent IL-1β-induced inflammation and cartilage degradation in osteoarthritis chondrocytes by directly binding to IL-1β and hindering the IL-1β–IL-1R1 interaction, thereby suppressing downstream MAPK and NF-κB activation [80].

MAPK signaling pathways (including ERK, JNK, p38) also play an important role in the pathogenesis of osteoarthritis. Traditional Chinese medicine and its active ingredients can treat osteoarthritis by inhibiting the abnormal activation of the MAPK signaling pathway [39,81]. Formononetin can reduce cartilage damage in osteoarthritis rats by down-regulating MAPK and NF-κB pathways [31].

Nrf2/Keap1 signaling pathway is the core regulatory system of cellular antioxidant defense. Plant-derived bioactive compounds can induce the expression of antioxidant enzymes such as heme oxygenase-1 (HO-1) and enhance the antioxidant capacity of cells by activating the Nrf2/Keap1 pathway [82].

NLRP3 inflammasome is an important component of the innate immune system, and its abnormal activation is closely related to the inflammatory response of osteoarthritis. The NLRP3 inflammasome drives IL-1β maturation and pyroptosis in chondrocytes and synovial macrophages. Chinese medicine monomers, extracts, and compounds can treat bone and joint diseases by interfering with the NLRP3 inflammasome [83]. For example, Resveratrol activates SIRT1, which deacetylates NLRP3 and suppresses its assembly. Similarly, berberine and andrographolide interfere with ASC oligomerization and caspase-1 activation. Beyond NLRP3, these compounds modulate macrophage polarization: eucommia polysaccharide reduces M1 (pro-inflammatory) macrophages while promoting M2 (anti-inflammatory) polarization, thereby reshaping the synovial immune microenvironment.

In addition, emerging evidence indicates that JAK/STAT signaling contributes to OA-related inflammation and cartilage catabolism. Certain flavonoids inhibit STAT3 phosphorylation, reducing SOCS3-mediated inflammatory amplification. Additionally, boswellic acid (specifically AKBA) and evodiamine exert dual inhibition on the COX-2 and 5-LOX pathways, suppressing prostaglandin E_2_ and leukotriene synthesis, which explains their potent analgesic and anti-edema effects in clinical settings. Therefore, the JAK/STAT pathway and the Cox/Lox pathway are also important targets for plant compounds to regulate the inflammatory response of arthritis [84].

### 4.2. Anti-Oxidative Stress

Oxidative stress is an important factor in the development of osteoarthritis. Excessive production of reactive oxygen species can directly damage chondrocytes and promote matrix degradation. Some medicinal and edible homologues with bioactive components, such as specific polyphenols (resveratrol, curcumin and EGCG), have shown strong antioxidant capacity. However, this property cannot be extended to all compounds in these categories.

Polyphenol compounds such as resveratrol, curcumin, and tea polyphenols are efficient active oxygen scavengers, which can directly neutralize free radicals and reduce oxidative damage. More importantly, they activate the Nrf2/Keap1/ARE axis. Upon stimulation, Nrf2 dissociates from Keap1, translocates to the nucleus, and upregulates antioxidant enzymes including heme oxygenase-1 (HO-1), superoxide dismutase (SOD), catalase (CAT), and glutathione peroxidase (GSH-Px). This transcriptional reprogramming enhances the long-term antioxidant capacity of chondrocytes rather than providing merely transient relief [78]. Betaine can alleviate osteoarthritis by inhibiting the production of reactive oxygen species and the MAPK signaling pathway [47].

In addition, mitochondria are also the main source of ROS in articular cartilage. Recent studies have shown that several MFH compounds regulate mitochondrial dynamics. They increase mitochondrial membrane potential, reduce mitochondrial ROS (mt ROS) leakage, and enhance mitophagy (selective autophagy of damaged mitochondria). By protecting the integrity of mitochondria, these compounds prevent the activation of ROS-induced downstream inflammation and apoptosis pathways, and establish a key link between energy metabolism and OA progression.

### 4.3. Regulating Chondrocyte Metabolism and Protecting Cartilage Matrix

The balance of synthesis and degradation of cartilage extracellular matrix is the basis for maintaining the normal function of articular cartilage. It has been reported that individual bioactive components in the pharmaceutical and food homology can regulate this balance through different mechanisms, depending on the particular compound under study.

MMPs (MMP-1, MMP-3, MMP-13) and ADAMTS-5 are the principal enzymes responsible for type II collagen and aggrecan degradation. Studies have shown that betaine can reduce the content of Col-X and MMP-13 in osteoarthritis chondrocytes. In ACLT-induced OA mice, betaine decreased the number of MMP-13-positive and collagen X-positive cells, prevented articular cartilage proteoglycan loss, and lowered OARSI scores [47,85]. Curcumin and resveratrol can reduce the expression of MMPs and ADAMTS by inhibiting NF-κB and MAPK signaling pathways, thereby slowing down the degradation of cartilage matrix [86].

Beyond inhibiting degradation, these compounds actively stimulate cartilage repair. Animal experiments showed that turmeric extract and pueraria coicis extract could promote the synthesis and secretion of cartilage collagen in osteoarthritis rats [48]. This anabolic effect may involve the activation of TGF-β/Smad signaling or the insulin-like growth factor-1 (IGF-1) pathway, although the precise molecular targets require further elucidation.

Chondrocyte apoptosis and hypertrophic differentiation accelerate cartilage loss. Betaine, isolated from Lycii Radicis Cortex, can attenuate osteoarthritis progression by inhibiting hyperactivated osteoclastogenesis and maintaining microarchitecture in subchondral bone. In ACLT-induced OA mice, betaine decreased the number of MMP-13-positive and collagen X-positive cells, prevented articular cartilage proteoglycan loss, lowered OARSI scores, normalized uncoupled subchondral bone remodeling (as defined by lowered trabecular pattern factor and increased subchondral bone plate thickness), and blunted aberrant angiogenesis in subchondral bone. Mechanistically, betaine suppressed osteoclastogenesis in vitro by inhibiting ROS production and subsequent MAPK signaling [47]. This effect suggests that medicinal and edible homologous components may play a comprehensive joint protection role by regulating bone-cartilage cross-talk.

### 4.4. Regulating Subchondral Bone Remodeling

OA is increasingly considered to be a disease of the whole joint organ, in which there is pathological crosstalk between subchondral bone and articular cartilage [87]. Some components of MFH/MHF have protective effects on both sides of the bone-cartilage interface.

Subchondral bone sclerosis and abnormal angiogenesis will aggravate cartilage degeneration and accelerate the development of osteoarthritis [88]. Betaine attenuates osteoclastogenesis and reduces subchondral bone angiogenesis by inhibiting ROS and MAPK pathways in osteoclast precursors. This prevents the formation of bone marrow lesions and reduces the transport of catabolic factors from bone to cartilage. *Eucommia ulmoides* polysaccharide increases subchondral cancellous bone density and trabecular thickness in rabbit OA models [46]. Geniposide and other iridoids from eucommia promote osteoblast proliferation and differentiation via the PI3K/Akt and Wnt/β-catenin pathways. By normalizing subchondral bone architecture, these compounds indirectly alleviate mechanical stress on overlying cartilage [89].

### 4.5. Autophagy, Ferroptosis, and Other Regulated Cell Death Pathways

Recent advances have highlighted alternative cell death mechanisms in OA beyond apoptosis.

Autophagy maintains chondrocyte homeostasis by degrading damaged organelles and misfolded proteins [90,91]. Berberine modulates autophagic flux through the AMPK/mTOR axis, preventing the accumulation of dysfunctional mitochondria and protein aggregates [38]. However, autophagy is a double-edged sword; excessive autophagy can lead to autophagic cell death. MFH compounds appear to restore physiologic autophagy levels rather than simply inducing or inhibiting the process [92].

Ferroptosis—an iron-dependent, lipid peroxidation-driven form of regulated cell death—has been implicated in OA pathogenesis [93]. *Lycium barbarum* polysaccharide alleviates OA progression in the destabilization of the medial meniscus (DMM) model by inhibiting chondrocyte ferroptosis, as evidenced by reduced lipid ROS and restored glutathione peroxidase 4 (GPX4) expression. This represents a novel mechanism distinct from traditional anti-apoptotic or anti-inflammatory actions [94].

### 4.6. Epigenetic Regulation

Recent studies have found that nutritional epigenetics plays an important role in the prevention and treatment of osteoarthritis. Plant-derived bioactive compounds can exert cartilage protection by regulating epigenetic modification.

Recent studies have found that nutritional epigenetics plays an important role in the prevention and treatment of osteoarthritis. Plant-derived bioactive compounds can exert cartilage protection by regulating epigenetic modification [95]. Specifically, polyphenols such as curcumin, ellagic acid, and EGCG can act as potent histone deacetylase (HDAC) inhibitors, preventing the removal of acetyl groups from lysine residues in histones and resulting in increased gene transcription [96]. Curcumin has been shown to induce enrichment of the H3K27ac mark in the promoter of superoxide dismutase 2 (Sod2), which is related to the upregulation of Sod2 and to an improved phenotype in inflammatory models [84]. Additionally, curcumin can regulate miRNA expression; for example, it upregulates miR-338-3p expression in chondrocytes, thereby inhibiting EIF4A1 and attenuating IL-1β-induced cytotoxicity and apoptosis [23]. These compounds can also regulate DNA methylation patterns, affect histone modifications (acetylation and methylation), and regulate chromatin remodeling of key inflammatory genes. The combination of natural epigenetic regulators is particularly important due to its additive and synergistic effects, safety and therapeutic efficacy, and fewer adverse reactions than traditional drugs in the treatment of osteoarthritis [95]. This emerging field has opened up a new perspective for the study of the anti-osteoarthritis mechanism of medicinal and edible homologous components.

### 4.7. Regulating Intestinal Flora

The gut–joint axis represents a frontier in OA research. Intestinal dysbiosis promotes systemic low-grade inflammation and metabolic endotoxemia, which can accelerate joint degeneration. Studies have shown that patients with osteoarthritis demonstrate a significant reduction in gut microbiota diversity and an imbalance in the proportions of specific microbial populations. Protective microbiota such as Bifidobacterium, Bacteroides, Faecalibacterium, and Roseburia are reduced, while pathogenic microbiota such as Streptococcus and Enterococcus are increased and positively correlated with OA pain and severity. A significant increase in the Firmicutes/Bacteroidetes (F/B) ratio indicates dysbiosis [97]. Intestinal flora imbalance may be involved in the development of osteoarthritis by promoting systemic inflammation and metabolic disorders. Mechanistically, dysbiosis compromises the intestinal epithelial barrier, allowing microbial products such as LPS to enter the systemic circulation. LPS binding to TLR4 on macrophages, dendritic cells, synovial fibroblasts, and chondrocytes triggers the MyD88–NF-κB signaling cascade, increasing the expression of COX-2, iNOS, IL-6, and TNF-α. Concurrently, microbial and danger-associated signals assemble the NLRP3 inflammasome, activating caspase-1 and converting pro-IL-1β and pro-IL-18 into their active forms, which amplify synovial inflammation and accelerate cartilage degradation [97,98].

From the perspective of balancing intestinal flora, screening small molecules of medicine and food homology for bone and joint protection and repair has become a new research idea. This direction provides a new dimension for the study of the anti-osteoarthritis mechanism of medicinal and edible homologous components.

*Lycium barbarum* polysaccharide increases the abundance of beneficial genera such as Lactobacillus while reducing pro-inflammatory taxa. This microbiota shift correlates with reduced serum LPS levels and decreased synovial inflammation. Short-chain fatty acids (SCFAs) produced by beneficial bacteria may mediate these systemic anti-inflammatory effects through G-protein-coupled receptors (GPRs) on chondrocytes and immune cells.

### 4.8. Interconnection and Synergy Between Mechanisms

Recent studies have shown that these mechanisms are not isolated; they form an integrated network.

NF-κB activation induces ROS production through NADPH oxidase, and ROS further activates NF-κB. Activation of Nrf2 not only enhances antioxidant defense, but also antagonizes NF-κB-driven inflammatory responses. This forms inflammation-oxidative coupling.

Mitochondrial dysfunction changes the NAD/NADH ratio, affects sirtuin activity, and thus affects histone acetylation patterns, leading to metabolic–epigenetic linkages.

Intestinal microbial metabolites affect macrophage polarization and Treg/Th17 balance, thereby regulating synovial inflammation and osteoclast formation and forming an intestinal–joint–immune triangle.

MFH/MHF bioactive components often target multiple nodes in the network at the same time, which explains their therapeutic advantages over single-target synthetic drugs. Future research should adopt multi-omics methods to systematically analyze these crosstalk mechanisms and determine the dominant pathways of specific OA subtypes.

## 5. Summary and Prospect

In summary, representative bioactive components from medicinal and health food sources have shown broad application prospects in the prevention and treatment of osteoarthritis. These components are rich in variety, covering polyphenols, flavonoids, terpenoids, alkaloids, polysaccharides, and other chemical types, and their mechanisms of action are diverse and compound-specific. Individual compounds may exert anti-oxidative effects, regulate chondrocyte metabolism, protect the cartilage matrix, mediate epigenetic regulation, or regulate the intestinal flora by regulating specific inflammatory signaling pathways (NF-κB, MAPK, Nrf2/Keap1, or NLRP3 in specific compounds), thereby exerting cartilage protection [40,77,99]. However, these mechanisms are targeted at specific bioactive compounds rather than the entire chemical class.

However, there are still many unsolved problems and challenges in this field. Firstly, many medicinal and edible homologous active ingredients (such as resveratrol, curcumin, etc.) have poor water solubility, limited intestinal permeability, and extensive first-pass metabolism, resulting in low and unstable in vivo exposure [100]. This severely restricts its clinical application. Future research should focus on the development of advanced delivery strategies such as nanocarriers, phytosomes, and liposome preparations to improve bioavailability and target tissue distribution [101]. Secondly, most current studies are still in the stage of cell and animal experiments, and high-quality randomized controlled clinical trials are lacking. The lack of standardization of botanical preparations and the inconsistency of clinical studies have hindered their wider clinical application. In the future, more well-designed clinical trials are needed to verify the efficacy and safety of the anti-osteoarthritis components [102]. Although a large number of studies have shown that medicinal and edible homologous components can play a role through multiple signaling pathways, the cross-talk between these pathways, synergistic effects, and differential effects in different osteoarthritis subtypes still need to be further elucidated [103]. In particular, the research on emerging mechanisms such as epigenetic regulation and intestinal flora regulation is still in its infancy [97,104]. In addition, with the development of bioinformatics and systems biology, the prediction of medicinal and edible Chinese medicine based on the characteristic genes of osteoarthritis and the screening of active components have become a new research direction [73]. In the future, chemical fingerprint, network pharmacology, molecular docking, and multi-omics technology should be combined to systematically carry out the screening, optimization, and mechanism research of active ingredients from medicine and food [105].

In summary, the bioactive components derived from medicinal and edible sources and medicinal health foods have important research value and development prospects as natural, safe, and multi-target anti-osteoarthritis candidates. With the deepening of research and the application of new technologies, these resources derived from traditional wisdom are expected to provide more effective solutions for the prevention and treatment of osteoarthritis.

## Figures and Tables

**Table 1:** Anti-osteoarthritis effect and mechanism of representative active ingredients in medicinal and edible homologous and health food.

Classification	Chemical Structure	Active Ingredient	Source (Representative Variety)	Experimental Model	Main Efficacy	Mechanism of Action	References
Medicine–Food Homology	Polyphenol compounds	Curcumin	Common turmeric rhizome	OA rats/chondrocytes	Reduce cartilage damage and promote collagen synthesis	Inhibition of NF-κB, MAPK pathway; reduces MMP-1/3/13; and upregulates type II collagen	[23,24]
		Resveratrol	Grapes, *Polygonum cuspidatum*	OA mouse/human chondrocytes	Anti-apoptosis, anti-inflammatory, delay cartilage degeneration	Activation of SIRT1 inhibits NF-κB and NLRP3 inflammasome	[25,26]
		Epigallocatechin gallate (EGCG) (tea polyphenols)	Green tea	OA rats/chondrocytes	Inhibition of IL-1β-induced inflammation and matrix degradation	Inhibition of AP-1, NF-κB activity, reduce MMP-13 and ADAMTS-5	[27,28]
	Flavonoids	Quercetin	Hawthorn, onion, *Sophora japonica*	OA chondrocyte	Anti-inflammatory, anti-oxidation, inhibition of chondrocyte apoptosis	Down-regulation of NF-κB and MAPK, activation of Nrf2/HO-1	[29,30]
		Formononetin	*Astragalus*, licorice	OA rats	Reduces cartilage damage and synovitis	Down-regulation of MAPK and NF-κB pathways	[31]
		Puerarin	Lobed kudzu vine root	OA rats	Inhibition of cartilage matrix degradation	Inhibition of ERK1/2 and p38 phosphorylation reduced MMP expression	
		Cyanidin	Berries, purple sweet potato	OA chondrocyte	Reduces ROS levels and inhibits inflammation	Scavenging free radicals and inhibiting COX-2 and iNOS	[32]
	Terpenoids	Boswellic acid (AKBA)	Olibanum	OA patients (clinical)	Improves pain and joint function	Inhibits 5-LOX and leukotriene synthesis; reduces TNF-α, IL-6	[33,34]
		Andrographolide	Creat	OA mice	Anti-inflammatory, protect cartilage	Blocking NF-κB nuclear translocation and inhibiting MMP activity	[35]
		Celastrol	*Tripterygium wilfordii*	OA models	Inhibits cartilage destruction, reduces pro-inflammatory factors	Suppresses NF-κB, activates autophagy	[36,37]
	Alkaloids	Berberine	*Coptis*, *Phellodendron*	OA rats	Anti-inflammatory, inhibition of synovial fibrosis	Inhibition of MAPK/NF-κB, down-regulation of IL-1β and TNF-α	[38,39]
		Sinomenine	*Sinomenium acutum*	OA rabbits/patients	Reduce IL-1β, PGE2, improve joint function	Inhibit COX-2, mPGES-1, NF-κB and JAK/STAT3	[40,41]
		Evodiamine	*Evodia rutaecarpa*	OA chondrocytes/mouse DMM	Reduce NO, IL-6, TNF-α, PGE2, inhibit MMP-13	Suppress NF-κB and p65 nuclear translocation	[42,43]
	Polyose	*Lycium barbarum* polysaccharide	Chinese matrimony vine	OA rats	Antioxidation, promotes chondrocyte proliferation	Up-regulates SOD and GSH-Px, reduces MDA, activates PI3K/Akt	[44,45]
		*Eucommia ulmoides* polysaccharide	Eucommia bark	OA rats	Improves cartilage structure and reduces chondrocyte apoptosis	Regulates the ratio of Bcl-2/Bax and inhibits Caspase-3	[46]
	Miscellaneous	Betaine	Wolfberry, beet	OA chondrocyte	Reduces Col-X and MMP-13, inhibits osteophyte formation	Inhibits ROS and MAPK pathways, and acts on osteoclasts and angiogenesis	[47]
Medicinal and Edible Compound and Extract	Turmeric + *Pueraria* *lobata* + coix seed extract	Turmeric, Pueraria, coix seed	—	OA rats	Promotes cartilage collagen synthesis and secretion, repairs joints	Synergistic anti-inflammatory effect and anti-oxidation	[48]
	Jiuwei Jianbu Drink	*Eucommia, Achyranthes,* Fructus *Psoraleae*, etc.	—	OA rats	Reduces oxidative stress and inflammatory damage	Mediates HMGB1/RAGE/PI3K/Akt pathway	[49,50]
	Eucommia water extract	eucommia bark	—	OA rats	Cartilage protection, improves cartilage metabolism	Regulates the balance of ECM degradation and synthesis	[46,51]
	Frankincense + Uncaria compound preparation	*Olibanum, Uncaria*	—	OA patients (clinical patients)	Relieves pain and improves WOMAC score	Dual inhibition of COX/LOX pathway	
Medicinal health food	*Boswellia carteri* Birdw.	—	Frankincense resin, functional beverages	Clinical studies	Anti-inflammatory, analgesic	Frankincense acid inhibits 5-LOX and reduces inflammatory factors	
	*Harpagophytum* *procumbens*	—	Gouguocao tea, tablets	Clinical/animal	Anti-inflammatory, analgesic, inhibition of COX-2	Uncaria extract (harpagoside) inhibits COX-2 and NF-κB	
	*Zingiber officinale* Roscoe	—	Ginger seasoning, ginger tea	Clinical/animal	Anti-inflammatory, inhibition of NF-κB	Gingerol inhibits NF-κB and reduces pro-inflammatory cytokines	
	*Cinnamomum cassia* Presl	—	Cinnamon powder, spices	Clinical/animal	Anti-inflammatory, antioxidant	Cinnamaldehyde and polyphenols modulate NF-κB and Nrf2 pathways	

## Data Availability

No new data were created or analyzed in this study. Data sharing is not applicable to this article.
